# RETRACTION: LINC01224 accelerates malignant transformation via MiR‐193a‐5p/CDK8 axis in gastric cancer

**DOI:** 10.1002/cam4.7337

**Published:** 2024-05-30

## Abstract

**RETRACTION**: H. Sun, J. Yan, G. Tian, X. Chen, W. Song, “LINC01224 accelerates malignant transformation via MiR‐193a‐5p/CDK8 axis in gastric cancer,” *Cancer Medicine* 10, no. 4 (2021): 1377‐1393, https://doi.org/10.1002/cam4.3726.

The above article, published online on 2 March 2021, in Wiley Online Library (wileyonlinelibrary.com), has been retracted by agreement between the journal Editor‐in‐Chief, Stephen Tait, and John Wiley and Sons Ltd. Following publication, concerns were raised by third parties regarding the origin of figure 10A. Several scientific irregularities and unexpected similarities between an instrument in this figure and figures from other papers by unrelated author groups were found. The authors had initially contacted the journal requesting to withdraw their article due to errors presented in their data. However, the authors did not provide further details and did not respond to our invitation to comment on the additional concerns raised, or provide the original, unmodified figures. The editors have lost confidence in the data and conclusions of this study.

**RETRACTION**: H. Sun, J. Yan, G. Tian, X. Chen, W. Song, “LINC01224 accelerates malignant transformation via MiR‐193a‐5p/CDK8 axis in gastric cancer,” *Cancer Medicine* 10, no. 4 (2021): 1377‐1393, https://doi.org/10.1002/cam4.3726.

The above article, published online on 2 March 2021, in Wiley Online Library (wileyonlinelibrary.com), has been retracted by agreement between the journal Editor‐in‐Chief, Stephen Tait, and John Wiley and Sons Ltd. Following publication, concerns were raised by third parties regarding the origin of figure 10A. Several scientific irregularities and unexpected similarities between an instrument in this figure and figures from other papers by unrelated author groups were found. The authors had initially contacted the journal requesting to withdraw their article due to errors presented in their data. However, the authors did not provide further details and did not respond to our invitation to comment on the additional concerns raised, or provide the original, unmodified figures. The editors have lost confidence in the data and conclusions of this study.

